# Age and Muscle Function Are More Closely Associated With Intracellular Magnesium, as Assessed by ^31^P Magnetic Resonance Spectroscopy, Than With Serum Magnesium

**DOI:** 10.3389/fphys.2019.01454

**Published:** 2019-11-27

**Authors:** Donnie Cameron, Ailsa A. Welch, Fatemeh Adelnia, Christopher M. Bergeron, David A. Reiter, Ligia J. Dominguez, Nicholas A. Brennan, Kenneth W. Fishbein, Richard G. Spencer, Luigi Ferrucci

**Affiliations:** ^1^Translational Gerontology Branch, Intramural Research Program, National Institute on Aging, National Institutes of Health, Baltimore, MD, United States; ^2^Norwich Medical School, University of East Anglia, Norwich, United Kingdom; ^3^Laboratory of Clinical Investigation, Intramural Research Program, National Institute on Aging, National Institutes of Health, Baltimore, MD, United States; ^4^Emory University School of Medicine, Atlanta, GA, United States; ^5^Department of Internal Medicine and Geriatrics, University of Palermo, Palermo, Italy

**Keywords:** magnesium, skeletal muscle, ^31^P magnetic resonance spectroscopy, aging, muscle strength, sarcopenia

## Abstract

Total serum magnesium is a common clinical measurement for assessing magnesium status; however, magnesium in blood represents less than 1% of the body’s total magnesium content. We measured intramuscular ionized magnesium by phosphorus magnetic resonance spectroscopy (^31^P-MRS) and tested the hypothesis that this measure better correlates with skeletal muscle function and captures more closely the effect of aging than the traditional measure of total serum magnesium. Data were collected from 441 participants (age 24–98 years) in the Baltimore Longitudinal Study of Aging (BLSA), a study of normative aging that encompasses a broad age range. Results showed that intramuscular ionized magnesium was negatively associated with age (β = −0.29, *p* < 0.001, *R*^2^ = 0.08) and positively associated with knee-extension strength (β = 0.31, *p* < 0.001, and *R*^2^ = 0.1 in women; and β = 0.2, *p* = 0.003, and *R*^2^ = 0.04 in men), while total serum magnesium showed no association with age or strength (*p* = 0.27 and 0.1, respectively). Intramuscular ionized magnesium was significantly lower in women that in men (*p* < 0.001), perhaps due to chronic latent Mg deficiency in women that is not otherwise detected by serum magnesium levels. Based on these findings, we suggest that intramuscular ionized magnesium from^ 31^P-MRS is a better clinical measure of magnesium status than total serum magnesium, and could be measured when muscle weakness of unidentified etiology is detected. It may also be used to monitor the effectiveness of oral magnesium interventions, including supplementation.

## Introduction

**FIGURE 1:**
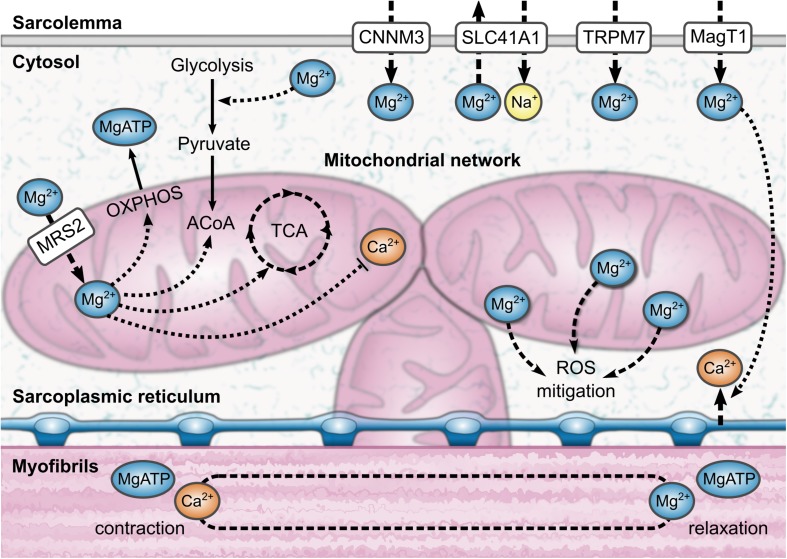
An illustration of the aspects of intramuscular magnesium physiology that can be observed using phosphorus magnetic resonance spectroscopy (^31^P-MRS). MRS is sensitive to the influence of magnesium complexation on the adenosine triphosphate molecule; this information can be used to estimate the bound magnesium fraction, and thus the proportion of free ionized magnesium, Mg^2+^. In brief, magnesium is involved in transport across the sarcolemma, energetic metabolism, and muscle contraction and relaxation. A third of the total magnesium content of a muscle cell can be found inside mitochondria. Magnesium also mitigates reactive oxygen species (ROS) in the cell through several mechanisms. ACoA, acetyl coenzyme A; CNNM3, cyclin M3; MagT1, magnesium transporter 1; MRS2, mitochondrial RNA splicing 2; OXPHOS, oxidative phosphorylation; SLC41A1, solute carrier family 41 type 1; TCA, tricarboxylic acid cycle; TRPM7, transient receptor potential melastatin type 7.

Although readily accessible as a clinical measurement, serum magnesium (Mg) represents only 0.3% of total body Mg, the majority of which is located in tissue and is active in a variety of enzymatic processes. The intramuscular Mg pool, illustrated in Figure 1, contains around 27% of the body’s Mg, second only to bone, which contains approximately 60% (Vormann, 2003). Mg in skeletal muscle and bone contributes to homeostasis (Lim et al., 1969), and defects in serum Mg may not become apparent until these stores are depleted (De Baaij et al., 2015). Indeed, some individuals show serum Mg levels within the reference range, but have a deficit in total body Mg, while others demonstrate low serum Mg levels but have a physiologic Mg content in tissue (Elin, 2010). Both hypomagnesemia and hypermagnesemia have traditionally been diagnosed by serum assays (Durlach, 1988); however, serum measures alone may not capture chronic latent Mg deficiency and depauperated Mg reserves (Elin, 2001, 2011), and both conditions may have important consequences for health (DiNicolantonio et al., 2018). Indeed, recent reports indicate that many Americans, especially older persons, do not consume the recommended daily amount of Mg: 420 mg for men and 320 mg for women (Institute of Medicine Standing Committee on the Scientific Evaluation of Dietary Reference Intakes, 1997; Vaquero, 2002; Ford and Mokdad, 2003; King et al., 2005). It has been suggested that inadequate Mg intake contributes to the loss of skeletal muscle mass and strength observed in most aging individuals and often associated with disability and higher mortality (Morley et al., 2001; Cruz-Jentoft et al., 2018). Indeed, recent work by Welch et al. has shown associations between dietary Mg and indices of skeletal muscle mass, grip strength, and leg explosive power in men and women of all ages (Welch et al., 2016, 2017; Hayhoe et al., 2018). For this reason, measures of intramuscular Mg may serve as better markers than serum Mg for understanding the relationship between Mg levels and skeletal muscle function.

Clinical assays for serum Mg give a “total” Mg concentration comprised of protein-bound, complexed, and ionized moieties, which leads to low sensitivity to the biologically active form of Mg, the ion Mg^2+^. Serum ionized Mg can be determined using ion-selective electrodes, but this technique cannot be used to measure intracellular Mg in tissue non-invasively. Furthermore, while 24-h urine Mg is useful (Witkowski et al., 2011), it is difficult to apply either in an individual subject or at the population level, and it may not reflect tissue Mg in non-steady-state conditions, such as acute kidney injury. Phosphorus magnetic resonance spectroscopy (^31^P-MRS) represents a promising alternative to these methods, providing a non-invasive *in vivo* measure of intracellular ionized Mg that can be applied directly in skeletal muscle (Williams et al., 1993; Iotti et al., 2000). The frequency shift of the β-adenosine-triphosphate (ATP) peak, as determined by ^31^P-MRS, depends on Mg complexation with ATP (Gupta et al., 1978), and gives an indirect estimate of free intracellular [Mg^2+^] when combined with a simultaneous measurement of pH from the ^31^P spectrum. Furthermore, ^31^P-MRS also offers insights into cell membrane metabolism and mitochondrial function and may shed light on how these processes change in dysmagnesemia.

The primary aim of this study is to test the hypothesis that intramuscular ionized Mg assessed by ^31^P-MRS is a better measure than total serum Mg for assessing Mg status, as well as to understand how Mg status changes with age and how it affects muscle function, namely knee-extension strength. To this end, we investigate the relationships between these measures, age, sex, and muscle function in a cohort of normatively aging men and women from the Baltimore Longitudinal Study of Aging (BLSA), ranging from 24 to 98 years old. A secondary aim is to investigate other ^31^P-MRS metabolites, such as phosphodiesters (PDEs), as possible non-invasive markers of cell damage in skeletal muscle in the context of dysmagnesemia.

## Materials and Methods

### Study Population

The BLSA is a study of human aging that was founded in 1958 and is currently conducted by the Intramural Research Program of the National Institute on Aging (Shock et al., 1984). Healthy volunteers of at least 20 years of age are continuously enrolled in the study and followed up at 1-to-4-year intervals, with follow-up visits being more frequent for older persons. At enrollment, participants must pass a comprehensive health and functional screening evaluation and be free of major chronic conditions and cognitive and functional impairments.

In this work, baseline data were obtained from 512 BLSA participants between August 2013 and January 2018 (235 men; median age = 73, range = 24–98 years), of whom 441 participants (210 men) had complete datasets, including a physical examination, ^31^P-MRS data, serum measures, and isometric knee extensor strength tests. Trained and certified technicians administered all tests according to standard protocols. Body weight in kilograms, and height and waist circumference in centimeters were assessed in all participants, and BMI was calculated as weight in kilograms divided by the square of height in meters.

### Phosphorus Magnetic Resonance Spectroscopy

**FIGURE 2:**
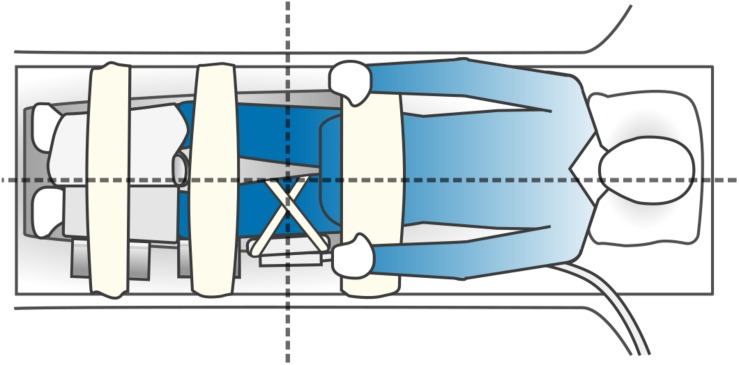
An illustration of participant setup for phosphorus magnetic resonance spectroscopy (^31^P-MRS). Subjects were positioned feet-first and supine in the bore of the MRI scanner, with a foam wedge under their knees and with the 10 cm ^31^P-MRS coil attached over their left vastus lateralis muscle using hook-and-loop straps. Participants were asked to shift to their right on the table to place the sensitive volume of the coil close to the magnet’s isocenter, which is indicated by the intersection of the dashed lines. A lower-leg positioning pad and a blanket were then placed over the shins as part of an exercise procedure performed subsequent to the rest MRS protocol detailed in this study. Wedges were placed at the left knee and shin to keep the left leg straight, and hook-and-loop straps and were affixed to the table over the hips, knees, and shins to limit gross displacements during the scan.

All ^31^P-MRS experiments were performed using a Philips Achieva 3.0T X-series MRI scanner (Philips Healthcare, Best, Netherlands) with a 10 cm transmit-receive loop coil tuned to phosphorus (PulseTeq, Surrey, United Kingdom). Data were acquired as part of a previously described exercise MRS protocol for assessing mitochondrial function (Choi et al., 2016; Zane et al., 2017); an illustration of the participant setup is shown in Figure 2. Briefly, subjects were positioned feet-first and supine with the loop coil placed over their vastus lateralis, midway between the greater trochanter and lateral femoral epicondyle. Participants were then shifted laterally to place the left thigh close to the magnet’s isocenter, and hook-and-loop straps and pads were applied to their hips, knees, and shins to limit gross displacements during the scan. After localizers and image-based shimming (Schär et al., 2004), and prior to the exercise MRS protocol, a static ^31^P spectrum was obtained from the entire sensitive volume of the loop coil with the following sequence parameters: repetition time = 25 s, spectral bandwidth = 2250 Hz, 2048 sampled points, 4 signal averages, 1 startup acquisition, and an adiabatic excitation pulse with a flip angle of 90°. A long repetition time was chosen to obviate the need for relaxation correction of metabolite ratios. The total exam time, including exercise MRS, was approximately 25 min.

#### Post-processing

Spectra were processed using an in-house software pipeline written in Python (version 3.5, Python Software Foundation^1^). This software performed 8 Hz Lorentzian apodization, two-times zero-filling, zero- and first-order phase correction, and shifting of the phosphocreatine peak to 0 ppm. Spectra were then automatically piped into the AMARES time-domain-fitting algorithm from jMRUI (version 3.0^2^; Vanhamme et al., 1997; Naressi et al., 2001; Stefan et al., 2009), which multiplied the first six points of each signal by a quarter-sine wave and corrected any residual phase errors before fitting all metabolite signals and calculating their amplitudes and chemical shifts. The chemical shifts of the two inorganic phosphate resonances, Pi_*a*_ and Pi_*b*_, were then used to calculate a weighted pH. First, the pH was calculated for each Pi resonance using the modified Henderson–Hasselbalch equation:

(1) $$\text{pH}=6.75+\log\frac{3.27-{\text{σ}}_{1}}{{\text{σ}}_{1}-5.69},$$

where σ_1_ is the chemical shift difference of the Pi peak relative to phosphocreatine. Second, the weighted pH was calculated as described by Reyngoudt et al. (2018):

(2) $${\text{pH}}_{\text{wt}}={\text{pH}}_{\text{a}}\cdot \frac{{\text{Pi}}_{\text{a}}}{{\text{Pi}}_{\text{tot}}}+{\text{pH}}_{\text{b}}\cdot \frac{{\text{Pi}}_{\text{b}}}{{\text{Pi}}_{\text{tot}}},$$

where pH_a_ is the pH determined for Pi_a_, pH_b_ is the pH for Pi_b_, and Pi_a_ and Pi_b_ are the amplitudes of those resonances, with Pi_tot_ as their sum. The concentration of free ionized intracellular Mg in muscle, [Mg^2+^] in millimolar units, was then estimated by using SciPy’s “newton” function to determine the roots of the following quadratic equation (Wary et al., 1999; Reyngoudt et al., 2019):

(3) $${\text{ δ }}_{\text{obs}}^{\alpha -\beta }=\frac{\begin{array}{ccc} {\text{ δ }}_{\text{ATP}}^{\alpha -\beta }+\delta_{\text{HATP}}^{\alpha -\beta }\cdot {\text{K}}_{\text{H}}\cdot \left[{\text{H}}^{+}\right]+{\text{ δ }}_{\text{MgATP}}^{\alpha -\beta }\cdot {\text{K}}_{\text{Mg}}\cdot & & \\ \left[{\text{Mg}}^{2+}\right]+{\text{ δ }}_{\text{MgHATP}}^{\alpha -\beta }\cdot {\text{K}}_{\text{H}}\cdot {\text{K}}_{\text{MgH}}\cdot \left[{\text{H}}^{+}\right]\,\left[{\text{Mg}}^{2+}\right]+ & & \\ {\text{ δ }}_{{\text{Mg}}_{2}\,\text{ATP}}^{\alpha -\beta }\cdot {\text{K}}_{{\text{Mg}}_{2}}\cdot {\text{K}}_{\text{Mg}}\cdot {\left[{\text{Mg}}^{2+}\right]}^{2} & & \end{array}}{\begin{array}{cc} 1+{\text{K}}_{\text{H}}\cdot \left[{\text{H}}^{+}\right]+{\text{K}}_{\text{Mg}}\cdot \left[{\text{Mg}}^{2+}\right]+{\text{K}}_{\text{H}}\cdot {\text{K}}_{\text{MgH}}\cdot & \\ \left[{\text{H}}^{+}\right]\,\left[{\text{Mg}}^{2+}\right]+{\text{K}}_{{\text{Mg}}_{2}}\cdot {\text{K}}_{\text{Mg}}\cdot {\left[{\text{Mg}}^{2+}\right]}^{2} & \end{array}},$$

where ${\text{δ}}_{\text{obs}}^{\alpha -\beta }$ is the observed chemical shift of β-ATP relative to α-ATP, [H^+^] = 10^−*pH*_*wt*_^, and formation constants K and chemical shifts δ are values taken from the literature (Table 1; Halvorson et al., 1992).

**TABLE 1:** Chemical shifts and formation constants used for the calculation of ionized intramuscular magnesium.

**Chemical shift (ppm)**	**Formation constant**
${\text{δ}}_{\text{ATP}}^{\alpha -\beta }$ = 10.78	K_H_ = 1.16 × 10^7^
${\text{δ}}_{\text{HATP}}^{\alpha -\beta }$ = 11.92	K_Mg_ = 4.86 × 10^4^
${\text{δ}}_{\text{MgATP}}^{\alpha -\beta }$ = 8.22	K_Mg2_ = 40
${\text{δ}}_{{\text{Mg}}_{2}\,\text{ATP}}^{\alpha -\beta }$ = 9.16	K_MgH_ = 5.98 × 10^2^
${\text{δ}}_{\text{MgHATP}}^{\alpha -\beta }$ = 8.52	

*Values are taken from Halvorson et al. (1992). All chemical shifts are relative to phosphocreatine and formation constants are given for a temperature of 37°C.*

The ratio of the PDE and γ-ATP resonance amplitudes was also calculated as an index of cell membrane damage. The PDE signal contains contributions from glycerol 3-phosphocholine and glycerol 3-phosphoethanolamine, which are thought to relate to membrane phospholipid breakdown (Ruiz-Cabello and Cohen, 1992).

### Laboratory Tests

Blood samples were obtained from participants after an overnight fast, between the hours of 07:00 and 08:00. Serum Mg, serum calcium, and serum albumin assays were performed on a Dimension Vista 1500 system (Siemens Healthcare Diagnostics, Tarrytown, NY, United States); total serum Mg and total serum calcium concentrations, in mg/dL, were measured using a conventional clinical colorimetric assay with bichromatic endpoints, and total serum albumin, in g/dL, was determined using a polychromatic endpoint method. Serum concentrations of 25-hydroxyvitamin D, in ng/mL, were determined using a chemiluminescent immunoassay on a LIAISON^®^ analyzer (DiaSorin Inc., Stillwater, MN, United States). Concentrations were converted from conventional units to SI units through multiplication by the following conversion factors: 0.41 for serum Mg, 0.25 for serum calcium, 0.1 for serum albumin, and 2.5 for 25-hydroxyvitamin D.

### Muscle Strength

Maximum quadriceps muscle strength was defined as the highest of three consecutive values of torque (N⋅m) measured by left-leg knee extensor contraction at a knee flexion of 70° using an isokinetic dynamometer (Biodex Multi-Joint System-PRO with Advantage Software Version 4X, Biodex Medical Systems, Inc., Shirley, NY, United States). Torque was determined as the force generated during knee-extension multiplied by the distance from the center of the knee to the point where the dynamometer was applied to the tibia.

### Statistics

All statistical analyses were performed in R (Version 3, R Foundation for Statistical Computing, Vienna, Austria). Intramuscular ionized Mg and total serum Mg data were divided into quartiles for illustration of their differing distributions. Cross-sectional relationships were evaluated with linear regression, where standardized variables were used to permit comparison of effect size, defined as the slope, β, of the regression. Nested linear regression models were fitted to test the association of intramuscular ionized Mg from ^31^P-MRS with knee-extension strength (Model 1) in male and female subgroups. Covariates included age, sex, and BMI (Model 2); and serum calcium, serum albumin, and serum 25-hydroxy-vitamin D (Model 3). Data were tested for normality using the Shapiro–Wilk test, and differences between groups were assessed with two-sided Student’s *t*-tests if data were normally distributed, or Mann–Whitney *U* tests if they were not. Differences in proportions were assessed using a chi-square test. A *p-*value less than 0.05 was considered statistically significant in all analyses.

### Study Approval

Ethical approval for this study was granted by the Institutional Review Board of the National Institute of Environmental Health Sciences. Each participant received a comprehensive description of the study, including possible risks, and gave written informed consent prior to their inclusion in the study, in accordance with the Declaration of Helsinki.

## Results

**FIGURE 3:**
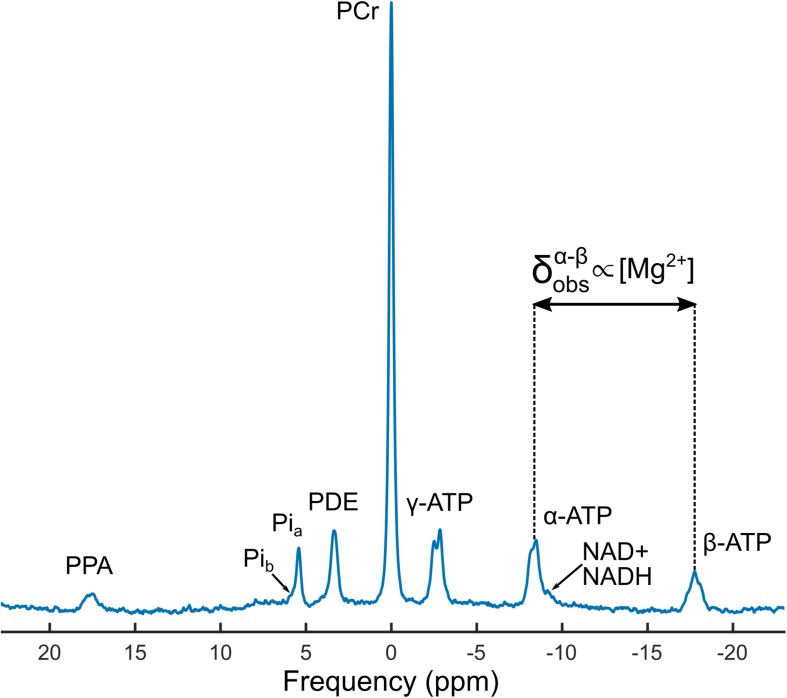
A representative phosphorus magnetic resonance spectroscopy (^31^P-MRS) spectrum from a 48-year-old male participant. The concentration of intramuscular ionized magnesium, [Mg^2+^], was determined using the observed chemical shift difference between the alpha and beta adenosine triphosphate (ATP) peaks, ${\text{δ}}_{\text{obs}}^{\alpha -\beta }$, along with the weighted pH calculated from the amplitudes and chemical shifts of inorganic phosphate peaks Pi_*a*_ and Pi_*b*_. Also shown are spectral lines from phosphocreatine (PCr), phosphodiesters (PDEs), oxidized and reduced nicotinamide adenine dinucleotide (NAD^+^ and NADH, respectively), and an external reference peak from phenylphosphonic acid (PPA).

Demographic details of the study cohort are summarized in Table 2, and a representative ^31^P MRS spectrum is shown in Figure 3. Quartiles of ^31^P-MRS-measured free ionized Mg in muscle and total serum Mg are summarized for various subgroups in Table 3 to illustrate the different distributions of these two Mg measures.

**TABLE 2:** Demographic data.

	**All (*n* = 441)**	**Female (*n* = 231, 52.3%)**	**Male (*n* = 210, 47.7%)**	***p-*value**
Age (years)	73 [16.3]	71.6 [16.5]	75.5 [16.7]	0.06
African–American race, *N* (%)	114 (25.9)	72 (31.2)	42 (20)	0.005^*†^
Caucasian race, *N* (%)	295 (66.9)	138 (59.7)	157 (74.8)	0.27^†^
Height (cm)	167.9 [13.8]	160.8 [8.6]	174.5 [8.7]	< 0.001^∗^
Weight (kg)	74.5 [20.2]	65.7 [17.8]	81.9 [17.2]	< 0.001^∗^
BMI (kg/m^2^)	26.0 [4.9]	25.2 [5.5]	26.6 [4.3]	< 0.001^∗^
Knee-extension torque (N⋅m)	120.9 [60.7]	104 [38.5]	149.5 [62.2]	< 0.001^∗^
Serum magnesium (mM)	0.78 [0.08]	0.78 [0.08]	0.78 [0.12]	0.55
Serum calcium (mM)	2.23 [0.1]	2.25 [0.13]	2.23 [0.13]	< 0.001^∗^
Serum albumin (g/L)	0.37 [0.04]	0.37 [0.03]	0.37 [0.03]	0.19
Serum 25-hydroxyvitamin D (nM)	87.5 [35]	90 [35]	85 [35]	0.004^∗^
Muscle ionized magnesium (mM)	0.55 [0.12]	0.52 [0.1]	0.59 [0.12]	< 0.001^∗^
Muscle pH	7.09 (0.04)	7.09 (0.04)	7.09 (0.03)	0.7
Muscle PDE-ATP ratio	0.73 [0.22]	0.72 [0.22]	0.74 [0.22]	0.19

*Demographic data for the participants included in this study, including serum measures and parameters derived from muscle ^31^P magnetic resonance spectroscopy. Data are expressed as mean (standard deviation) or median [interquartile range], except where noted, and differences between men and women were assessed using Student’s *t*-tests or Mann–Whitney *U* tests, respectively. ATP, adenosine triphosphate; PDE, phosphodiesters; ^∗^statistically significant; *^†^*chi-squared test *p*-value.*

**TABLE 3:** Quartiles of intramuscular ionized magnesium and total serum magnesium.

	**Muscle ionized magnesium, mM**			**Total serum magnesium, mM**		
		
	**Quartile 1**	**Quartile 2**	**Quartile 3**	**Quartile 1**	**Quartile 2**	**Quartile 3**
All participants (*n* = 441)	0.498	0.552	0.622	0.738	0.779	0.82
Female (*n* = 231, 52.3%)	0.48	0.52	0.578	0.738	0.779	0.82
Male (*n* = 210, 47.7%)	0.534	0.593	0.651	0.738	0.779	0.861
Age 24–40 years (*n* = 19, 4.3%)	0.568	0.635	0.689	0.738	0.82	0.82
Age 40–60 years (*n* = 51, 11.6%)	0.564	0.595	0.652	0.738	0.779	0.82
Age 60–80 years (*n* = 239, 54.2%)	0.5	0.55	0.624	0.738	0.779	0.82
Age > 80 years (*n* = 132, 29.9%)	0.471	0.53	0.581	0.738	0.82	0.861
African–American (*n* = 114, 25.9%)	0.497	0.567	0.626	0.697	0.738	0.82
Caucasian (*n* = 295, 66.9%)	0.496	0.549	0.61	0.738	0.779	0.861

*Data are provided for the whole cohort and subdivided by sex, age, and race.*

### Relationship Between Ionized Magnesium in Muscle and Total Serum Magnesium

Free cytosolic [Mg^2+^] in muscle showed a weak inverse association with total serum Mg, with β = −0.1, 95% CI = [−0.19, 0], *p* = 0.04, adjusted *R*^2^ = 0.01. This association remained significant after adjusting for serum calcium, serum albumin, and 25-hydroxyvitamin D, with β = −0.11, 95% CI = [−0.2, −0.02], *p* = 0.02, adjusted *R*^2^ = 0.08.

**FIGURE 4:**
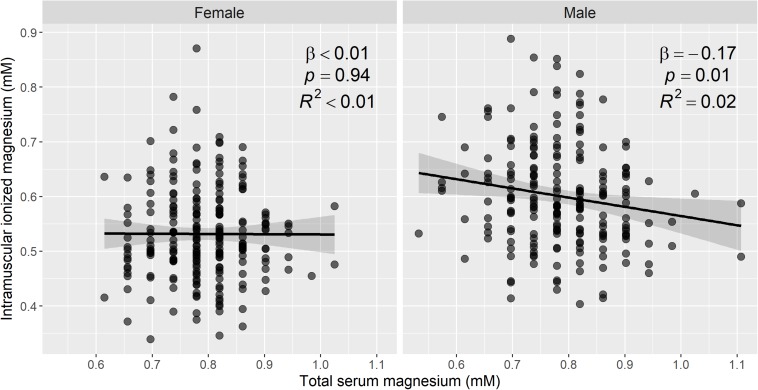
Intramuscular ionized magnesium is associated with total serum magnesium in men, but not in women. Sex-stratified scatter plots of the estimated ionized magnesium concentration in muscle, from ^31^P magnetic resonance spectroscopy, versus the total serum magnesium concentration in 441 participants. To convert mM concentrations of serum magnesium to mg/dL, divide by 0.411. Linear regression was used to assess the association between the two magnesium measures, and the regression line, 95% confidence intervals, and summary statistics are shown. There was a statistically significant negative association between intramuscular ionized magnesium and total serum magnesium in men, but not in women. A total of 459 participants had complete data for ^31^P-MRS muscle magnesium and serum magnesium.

After stratification by sex, there was no apparent association between total serum Mg and intramuscular [Mg^2+^] in women (*p* = 0.94), but there was an inverse association in men, with β = −0.17, 95% CI = [−0.3, −0.03], *p* = 0.01, adjusted *R*^2^ = 0.02. These data are illustrated by a scatter plot in Figure 4. Muscle pH values were not significantly different between the two sexes.

There was no statistically significant difference (*t*-test, *p* = 0.19) in intramuscular [Mg^2+^] between individuals who were hypomagnesemic (total serum Mg < 0.66 mM; *n* = 39, 8.8%) or hypermagnesemic (total serum Mg > 0.95 mM; *n* = 8, 1.8%).

### Myocellular Membrane Breakdown With Age and Its Association With Muscle Magnesium

The PDE-ATP ratio obtained from ^31^P-MRS is a marker of cell membrane breakdown. In our study this parameter showed a weak positive association with age, with β = 0.21, 95% CI = [0.12, 0.31], *p* < 0.001, adjusted *R*^2^ = 0.04. After including intramuscular [Mg^2+^] as a covariate in the model, the association between PDE:ATP and age remained significant (β = 0.26, 95% CI = [0.16, 0.35], *p* < 0.001), and intramuscular [Mg^2+^] was seen to be positively associated with PDE:ATP, with β = 0.15, 95% CI = [0.05, 0.24], *p* = 0.002, adjusted *R*^2^ = 0.06.

### Relationship Between Mg Measures and Age and Sex

**FIGURE 5:**
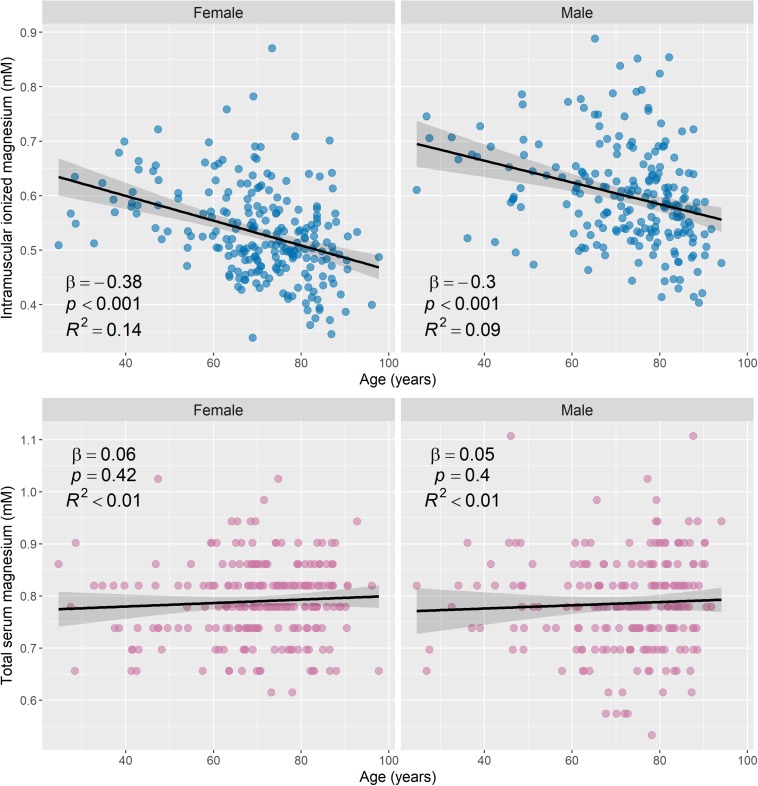
Intramuscular ionized magnesium is negatively associated with age, while total serum magnesium has no association with age. Scatter plots of the estimated ionized magnesium concentration in muscle versus age **(top row)** and total serum magnesium versus age **(bottom row)**, with stratification by sex (231 female and 210 male). Linear regression was used to assess associations between the magnesium measures and age, and regression lines, 95% confidence intervals, and summary statistics are shown for each subgroup. There was a statistically significant negative association between intramuscular ionized magnesium and age for male and female subgroups, but total serum magnesium was not significantly associated with age. To convert mM concentrations of serum magnesium to mg/dL, divide by 0.411.

Figure 5 shows scatter plots of muscle [Mg^2+^] and total serum Mg versus age, stratified by sex. There was an association between muscle [Mg^2+^] and age (β = −0.29, 95% CI = [−0.38, −0.2], *p* < 0.001, adjusted *R*^2^ = 0.08), which remained significant when men and women were assessed separately (Figure 2): β = −0.38, 95% CI = [−0.5, −0.26], *p* < 0.001, adjusted *R*^2^ = 0.14, for women; and β = −0.3, 95% CI = [−0.43, −0.17], *p* < 0.001, adjusted *R*^2^ = 0.09, for men. Total serum Mg was not significantly associated with age in the whole cohort (*p* = 0.27), or in male and female subgroups (*p* = 0.4 and 0.42, respectively).

**FIGURE 6:**
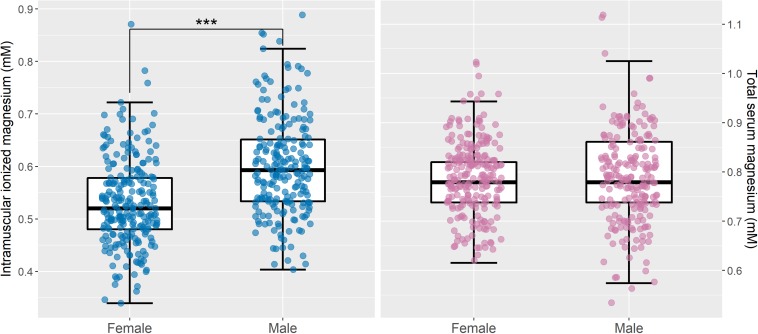
Intramuscular ionized magnesium is significantly lower in women than in men, but there are no sex differences in total serum magnesium. Boxplots of intramuscular ionized magnesium **(left)** and total serum magnesium **(right)** for male (*n* = 210) and female (*n* = 231) subgroups, with the raw data superimposed. Mann–Whitney *U* tests indicated that intramuscular ionized magnesium concentrations were significantly higher in men than in women (^∗∗∗^*p* < 0.001), but there was no statistically significant difference in total serum magnesium between the two groups (*p* = 0.37). After adjusting for age, intramuscular ionized magnesium remained significantly different between men and women (*p* < 0.001), and there was still no significant difference in total serum magnesium (*p* = 0.31). Convert mM concentrations of serum magnesium to mg/dL by dividing by 0.411.

Figure 6 shows boxplots of muscle [Mg^2+^] and total serum Mg categorized by sex. Intramuscular [Mg^2+^] was significantly higher in male participants than in female participants (*p* < 0.001), and this difference remained significant after adjusting for age (*p* < 0.001). To determine whether this was driven by known post-menopausal Mg changes in women (Stanton and Lowenstein, 1987), a Mann–Whitney *U* test was applied to compare intramuscular [Mg^2+^] between men and women under the age of 45; however, there was still a significant difference between the two groups (*p* = 0.017). Total serum Mg did not show a statistically significant difference with sex, either before adjusting for age (*p* = 0.55) or after (*p* = 0.3).

### Relationship Between Magnesium Measures and Muscle Strength

**FIGURE 7:**
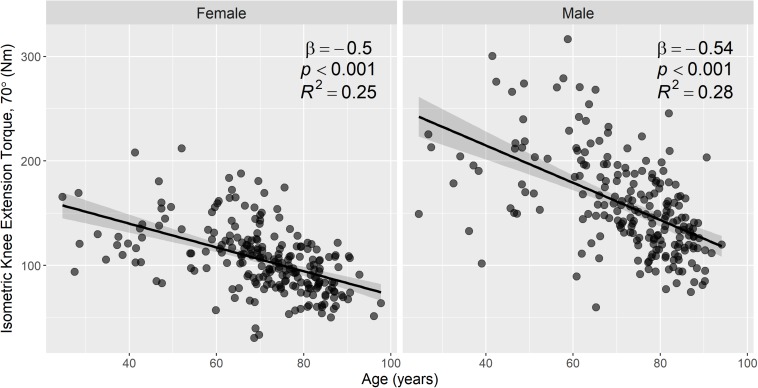
Knee-extension torque is strongly associated with age in both men and women. Scatter plots of knee-extension torque versus age, with stratification by sex (231 female, 210 male). Knee-extension torque was measured using isometric knee extensor contractions at a knee flexion of 70°. Associations between knee-extension torque and age were assessed by linear regression, and regression lines, 95% confidence intervals, and summary statistics are shown for each subgroup. There were statistically significant negative associations between knee-extension torque and age in both male and female subgroups.

**FIGURE 8:**
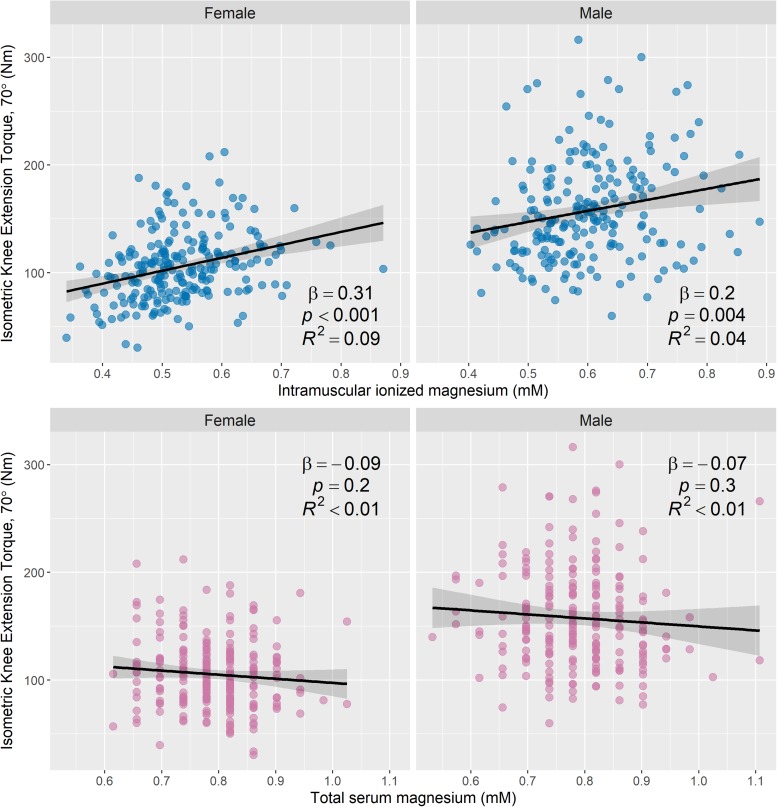
Intramuscular ionized magnesium is associated with knee-extension torque, but total serum magnesium is not. Scatter plots of knee-extension torque versus the estimated ionized magnesium concentration in muscle **(top row)** and total serum magnesium **(bottom row)**, with stratification by sex (231 female and 210 male). Knee-extension torque was measured using isometric knee extensor contractions at a knee flexion of 70°. Associations between knee-extension torque and magnesium measures were assessed using linear regression, and regression lines, 95% confidence intervals, and summary statistics are shown for each subgroup. There was a statistically significant positive association between knee-extension torque and intramuscular ionized magnesium for both male and female subgroups, but knee-extension torque was not significantly associated with total serum magnesium. Convert mM concentrations of serum magnesium to mg/dL by dividing by 0.411.

Figure 7 shows the relationship between knee-extension torque and age in male and female participants. Scatter plots of muscle [Mg^2+^] and total serum Mg versus knee-extension torque are shown in Figure 8, again categorized by sex. Table 4 shows nested models of regression for the association of intramuscular ionized Mg with knee-extension strength, split by sex: Model 1 shows standardized regression coefficients for the association of muscle [Mg^2+^] and knee-extension strength; Model 2 includes adjustment for age, race, and BMI; and Model 3 includes additional covariates that may influence Mg metabolism or muscle function: serum calcium, serum albumin, and serum 25-hydroxy-vitamin D. The association between muscle [Mg^2+^] and knee-extension strength remained statistically significant in women, after adjusting for these parameters, but not in men. Total serum Mg was not significantly associated with knee-extension torque in the whole cohort (*p* = 0.1) or separately in men (*p* = 0.3) and women (*p* = 0.2).

**TABLE 4:** Nested linear regression models.

**Female (*n* = 231, 52.3%)**						
	
	**Model 1: *R*^2^ = 0.1**		**Model 2: *R*^2^ = 0.31**		**Model 3: *R*^2^ = 0.3**	
	**β [95% CI]**	***p***	**β [95% CI]**	***p***	**β [95% CI]**	***p***
Muscle ionized magnesium (mM)	0.31 [0.19, 0.44]	<0.001^∗^	0.16 [0.04, 0.28]	0.008^∗^	0.17 [0.05, 0.29]	0.007^∗^
Age (years)	−	−	−0.44 [−0.56, −0.32]	<0.001^∗^	−0.44 [−0.57, −0.31]	<0.001^∗^
Race	−	−	<0.01 [−0.11, 0.11]	0.96	−0.01 [−0.12, 0.11]	0.91
BMI (kg/m^2^)	−	−	0.21 [0.1, 0.33]	<0.001^∗^	0.18 [0.06, 0.3]	0.003^∗^
Serum calcium (mM)	−	−	−	−	0.01 [−0.11, 0.14]	0.82
Serum albumin (g/L)	−	−	−	−	−0.04 [−0.17, 0.09]	0.55
Serum 25-Hydroxy-vitamin D (nM)	−	−	−	−	−0.06 [−0.18, 0.05]	0.29

**Male (*n* = 210, 47.7%)**						
	
	**Model 1: *R*^2^ = 0.04**		**Model 2: *R*^2^ = 0.3**		**Model 3: *R*^2^ = 0.31**	
	**β [95% CI]**	***p***	**β [95% CI]**	***p***	**β [95% CI]**	***p***

Muscle ionized magnesium (mM)	0.2 [0.07, 0.34]	0.003^∗^	0.02 [−0.1, 0.15]	0.69	0.02 [−0.1, 0.15]	0.69
Age (years)	−	−	−0.51 [−0.63, −0.39]	<0.001^∗^	−0.5 [−0.63, −0.36]	<0.001^∗^
Race	−	−	0.03 [−0.08, 0.15]	0.55	0.04 [−0.07, 0.16]	0.47
BMI (kg/m^2^)	−	−	0.16 [0.04, 0.28]	0.007^∗^	0.17 [0.05, 0.29]	0.006^∗^
Serum calcium (mM)	−	−	−	−	−0.11 [−0.25, 0.03]	0.11
Serum albumin (g/L)	−	−	−	−	0.08 [−0.08, 0.23]	0.33
Serum 25-Hydroxy-vitamin D (nM)	−	−	−	−	<−0.01 [−0.12, 0.12]	0.95

*These models test the association between intramuscular ionized magnesium and knee-extension strength for female (top section) and male (bottom section) participants, accounting for different sets of confounders. Model 1 tests the unadjusted association between intramuscular ionized magnesium and knee-extension strength; model 2 was adjusted for age, race, and body mass index (BMI); model 3 was adjusted for age, race, BMI, serum calcium, serum albumin, and serum 25-Hydroxyvitamin D. All regression coefficients, β, are standardized. ^∗^Statistically significant.*

## Discussion

This is the first study, to our knowledge, that investigates *in vivo* intramuscular Mg in a large cohort of participants with a broad age range. We show that our ^31^P-MRS-based measure of intramuscular ionized Mg captures the effects of age and sex on magnesium status and is an independent correlate of skeletal muscle function, and knee-extension strength, while a conventional clinical assay of total serum Mg was not correlated with these parameters. Our method represents a non-invasive alternative to muscle biopsies for assessing ionized Mg content in muscle. It may be of particular interest for: (1) examining cases of muscle dysfunction where poor Mg status is suspected and the search for other causes is negative; and (2) tracking the effectiveness of Mg supplementation or other interventions aimed at improving Mg status where the homeostatic regulation of Mg may not track with functional reserves.

Serum Mg is the most common measure of Mg status; however, it does not consistently correlate with tissue Mg concentrations (Fiser et al., 1998; Emelyanov et al., 1999), and this is true of the data shown here. More than 400 enzymatic processes depend on Mg, so maintaining stable serum levels is crucial. Total serum Mg concentrations range from around 0.7–1.05 mM in healthy people (Lowenstein and Stanton, 1986), and our data from the BLSA cover a similar range; however, in our cohort, seemingly hypomagnesemic participants with serum levels lower than 0.66 mM did not show a concomitant reduction in muscle function. It is now well-established that some persons who present with a normal serum Mg concentration may in fact be severely Mg deficient, or even in excess of Mg (Elin, 2010; De Baaij et al., 2015). Such individuals may have a chronically low dietary intake of Mg, which would lead to low Mg concentrations in muscle and bone as these pools are accessed to maintain normal serum levels. This tight homeostasis may explain the weak or non-existent association between total serum Mg and intramuscular ionized Mg demonstrated in previous work (Rosenstein et al., 1995; Carlier et al., 1996; Ryschon et al., 1996), as well as the weak association shown in this study.

We show slightly lower concentrations of ionized intramuscular Mg than Wary et al. (1999); however, participants in that work were all young males under the age of 35, while more than 75% of our cohort are over the age of 65, with many female participants. Indeed, our results show significantly lower ionized intramuscular Mg in women than in men, but there was no difference in total serum Mg between these two groups. Several studies have shown sex differences in total serum Mg concentrations (Lowenstein and Stanton, 1986; Ryschon et al., 1996; Hayhoe et al., 2018), though others have not (Dominguez et al., 2006). Work by Ryschon et al. (1996) showed statistically significant sex differences in both total serum Mg and ^31^P-MRS-measured intramuscular ionized Mg, where the former was lower in women and the latter was lower in men. In contrast, we found that that intramuscular ionized Mg was lower in women than in men; however, our cohort is much larger than the group studied by Ryschon et al., and the participants are substantially older. The significantly lower muscle Mg that we show in women may indicate a trend of chronic latent Mg deficiency in this study cohort that is not otherwise detected by total serum magnesium levels (Elin, 2001, 2011). This may also explain other observations from our data: (1) that there was no apparent association between total serum Mg and intramuscular [Mg^2+^] in women, while there was in men; and (2) that serum calcium levels were significantly higher in women than in men.

In this work, we sought to validate intramuscular ionized Mg as an alternative to total serum Mg using a measure of knee extension strength and, indeed, we found a statistically significant association between intramuscular [Mg^2+^] and knee-extension strength in our study cohort. However, after adjusting for age, race, and BMI, this association only remained significant in women and not in men. This marked sex difference may relate to the lower intramuscular Mg content we observed in women. In support of the association we demonstrate between intramuscular [Mg^2+^] and muscle strength, work by Niermann et al. (2002) has shown reduced intramuscular [Mg^2+^] and [MgATP] in adult and juvenile dermatomyositis, conditions associated with muscle weakness and fatigue. Dominguez et al. (2006) demonstrated strong associations between total serum Mg and muscle performance in the InCHIANTI study, where muscle performance included measures of grip strength, lower-leg muscle power, and knee- and ankle-extension strength. However, in the present work, total serum Mg was not associated with knee-extension strength, despite a strong association between our intramuscular [Mg^2+^] measure from ^31^P-MRS and muscle strength. This disparity may stem from differences between the two cohorts: BLSA participants tend to be very healthy relative to the general population, while InCHIANTI participants are more representative of the broader public, being selected at random from census records (Ferrucci et al., 2000).

Many older adults in the United States do not consume the recommended daily amount of Mg (Vaquero, 2002), likely due the progressive decrease in the Mg content of foods resulting from food processing and changes in eating habits (Marier, 1986). Our results show a decline in muscle function with age that may be a consequence of this inadequate Mg intake. Studies looking at the relationship between dietary Mg and muscle strength have shown mixed results: some reported associations between the two measures (Veronese et al., 2014; Welch et al., 2016, 2017), while others did not (Scott et al., 2010; Moslehi et al., 2013). A recent intervention study also found a significant effect of Mg supplementation on functional measures, and this effect was more pronounced in women with a low dietary intake of Mg (Veronese et al., 2014). Total serum Mg can be used as an integrated measure of dietary intake and other factors (Ayuk and Gittoes, 2014); however, it does not accurately reflect dietary intake. Our ^31^P-MRS measures of intramuscular ionized Mg may represent a much superior alternative to total serum Mg for assessing intake and monitoring supplementation, as well as offering valuable clinical insight into the etiology of muscle dysfunction. It will be of particular interest in studying sarcopenia, the loss of skeletal muscle mass and strength with age.

Regarding future work, Dominguez et al. highlighted three possible mechanisms for their observed association between total serum Mg and muscle strength: the role of Mg in cell energetics; increased oxidative stress in hypomagnesemia; and the pro-inflammatory effect of Mg deficiency. Magnesium’s role in muscle energy metabolism may be elucidated through the expansive BLSA dataset, which also includes ^31^P-MRS-derived measures of mitochondrial function (Choi et al., 2016; Fabbri et al., 2017; Zane et al., 2017). Reactive oxygen species and inflammation, and their relationship with intramuscular Mg, might be explored using other ^31^P-MRS measures, such as the PDE:ATP ratio, which is thought to reflect cell membrane breakdown. We showed that PDE:ATP was positively associated with age, suggesting greater membrane phospholipid breakdown in older age that may be related to oxidative stress. When ionized intramuscular Mg was included as a covariate, it was seen to be positively associated with PDE:ATP. This appears to contradict previous studies that show an association between Mg concentration and oxidative stress; however, a positive association between PDE:ATP and ionized intramuscular Mg may in fact reflect healthy membrane turnover that is facilitated by greater concentrations of intracellular Mg. For comparison, recent work by Reyngoudt et al. (2019) has shown a negative association between [Mg^2+^] and PDE:ATP that was hypothesized to relate to disruption of the cellular membrane by Ca^2+^. They also showed a negative correlation between [Mg^2+^] and the phosphomonoester-ATP ratio, which is thought to be a marker of membrane synthesis. The phosphomonoester resonance was not resolvable in our dataset; however, additional data could be acquired to explore the relationship between intramuscular Mg and membrane metabolism in greater detail.

We found that intramuscular ionized magnesium was associated with age and muscle function while total serum magnesium was not. As such, our ^31^P-MRS measure of muscle magnesium represents a superior assay of magnesium status to total serum magnesium. Thus, it shows potential for identifying chronic latent magnesium deficiency; for investigating muscle dysfunction, particularly in the context of sarcopenia and frailty; and for testing of dietary, exercise, and pharmaceutical interventions.

## Data Availability Statement

The raw data supporting the conclusion of this manuscript will be made available by the authors, without undue reservation, to any qualified researcher, provided they submit a research proposal on the Baltimore Longitudinal Study of Aging website: https://www.blsa.nih.gov/how-apply.

## Ethics Statement

The studies involving human participants were reviewed and approved by the Institutional Review Board of the National Institute of Environmental Health Sciences. The patients/participants provided their written informed consent to participate in this study.

## Author Contributions

DC, AW, FA, and LF conceived the study. DC, FA, CB, DR, NB, and KF acquired the data. DC analyzed the data, performed the statistical analysis, and drafted the manuscript. All authors contributed to the manuscript revision and read and approved its submitted version.

## Conflict of Interest

The authors declare that the research was conducted in the absence of any commercial or financial relationships that could be construed as a potential conflict of interest.

## Footnotes

1 www.python.org

2 www.jmrui.eu
